# Exploring interdependencies, vulnerabilities, gaps and bridges in care transitions of patients with complex care needs using the Functional Resonance Analysis Method

**DOI:** 10.1186/s12913-023-09832-7

**Published:** 2023-08-11

**Authors:** Ann-Therese Hedqvist, Gesa Praetorius, Mirjam Ekstedt

**Affiliations:** 1 Department of Health and Caring Sciences, Linnaeus University, Kalmar, Sweden; 2 Ambulance Service, Region Kalmar County, Västervik, Sweden; 3 Swedish National Road and Transport Research Institute, Linköping, Sweden; 4 Department of Maritime Operations, University of South-Eastern Norway, Borre, Norway; 5 Department of Learning, Informatics, Management and Ethics, LIME, Karolinska Institutet, Stockholm, Sweden

**Keywords:** Complexity, Care transitions, Hospital discharge, Patient safety, Resilience, Functional Resonance Analysis Method (FRAM)

## Abstract

**Background:**

Hospital discharge is a complex process encompassing multiple interactions and requiring coordination. To identify potential improvement measures in care transitions for people with complex care needs, intra- and inter-organisational everyday work needs to be properly understood, including its interdependencies, vulnerabilities and gaps. The aims of this study were to 1) map coordination and team collaboration across healthcare and social care organisations, 2) describe interdependencies and system variability in the discharge process for older people with complex care needs, and 3) evaluate the alignment between discharge planning and the needs in the home.

**Methods:**

Data were collected through participant observations, interviews, and document review in a region of southern Sweden. The Functional Resonance Analysis Method (FRAM) was used to model the discharge process and visualise and analyse coordination of care across healthcare and social care organisations.

**Results:**

Hospital discharge is a time-sensitive process with numerous couplings and interdependencies where healthcare professionals’ performance is constrained by system design and organisational boundaries. The greatest vulnerability can be found when the patient arrives at home, as maladaptation earlier in the care chain can lead to an accumulation of issues for the municipal personnel in health and social care working closest to the patient. The possibilities for the personnel to adapt are limited, especially at certain times of day, pushing them to make trade-offs to ensure patient safety. Flexibility and appropriate resources enable for handling variability and responding to uncertainties in care after discharge.

**Conclusions:**

Mapping hospital discharge using the FRAM reveals couplings and interdependencies between various individuals, teams, and organisations and the most vulnerable point, when the patient arrives at home. Resilient performance in responding to unexpected events and variations during the first days after the return home requires a system allowing flexibility and facilitating successful adaptation of discharge planning.

**Supplementary Information:**

The online version contains supplementary material available at 10.1186/s12913-023-09832-7.

## Background

Advancements in modern medicine and technology have led to new medical interventions and promote high-quality care, creating possibilities to effectively treat acute diseases and leading to a decline in mortality and increased life expectancy [1]. The advancements also lead to increased specialisation and differentiation which may create gaps in communication and knowledge transfer between different healthcare professions and care providers. Patients with chronic illnesses and complex care needs, requiring care from both healthcare and social care providers [2, 3], are especially vulnerable to fragmentation of care as they will meet different professionals working for several care providers, whether seeking help for chronic or acute conditions [4, 5]. Coordinating care between different care providers is recognised as difficult [6] and is complicated by the diversity of actors and organisations involved. Due to the burden of increased chronic illness in the aging population, healthcare systems worldwide experience challenges in providing safe and efficient care to older people with complex care needs [3, 7].

Healthcare can be classified as a complex adaptive system (CAS), as its components have multiple interdependencies and work in nonlinear ways [8, 9]. Complex systems have fuzzy boundaries and co-evolve with other systems [10]. A CAS has intrinsic laws or principles, such as self-organisation, emergent behaviour and the capacity to learn, making it difficult to predict how its components will interact temporally in a given situation [11]. This implies that the view of the horizon for each person in the system is quite limited – and the further away it is, the more unpredictable the consequences of any actions taken become. Safety problems are thus not necessarily a direct result of lacking knowledge or limited effort on the part of the healthcare professionals – rather, they are usually a result of the complexity of the system.

An illustrative example of complexity in healthcare is the multidisciplinary teamwork in coordinated discharge planning [12] and the coordination of care for older people with complex care needs. Achieving safe care transitions from hospital to home for patients with complex care needs is a complicated process requiring successful completion of a number of tasks, e.g., coordinating care with healthcare personnel outside hospital [13]. The structural gaps between services and settings involved in these care transitions create safety gaps [14] and a healthcare system design with highly specialised care units may raise walls that obscure a patient’s care pathway and hide safety risks [15]. In a CAS, the known and the unpredictable exist side by side. During discharge planning, this creates a need for adaptability and flexibility, where each system unit may need to relinquish some of its autonomy and favour teamwork over professional and organisational boundaries.

As outlined above, care transitions can be understood as processes in a CAS. To reflect this, care transitions can be analysed using systemic safety models that promote a focus not only on the individuals, but on the interactions between the people, the technology and the organisations involved. Systemic models aid the identification and understanding of potential vulnerabilities at the system level instead of focusing on individuals as the root causes of any failure (or ‘scapegoats’) [16]. However, there is often a mismatch between how work is presumed to be performed and how it is actually performed. This mismatch is also referred to as the discrepancy between work-as-imagined (WAI) and work-as-done (WAD) (16, 17). When the concepts of WAI and WAD are related to the framework of WAx [17], work may also be represented by the perspectives of work-as-prescribed (in procedures and guidelines), work-as-normative (in rules or laws), work-as-observed (by the analyst), and work-as-disclosed (by the sharp-end or blunt-end operator). Depending on the dimension being investigated, as well as the perspective used for the analysis, work may thus be represented differently [18]. Regardless of this, when performance of tasks is adapted to changing operational conditions across organisations, such as constraints in time or resources, variability will occur due to trade-offs.

Poor healthcare quality is often related to the inherent complexity of the healthcare system, characterised by silos, multiple stakeholder interactions and a significant degree of performance variability within and across system levels [19–21]. Care transitions, especially hospital discharges, are complex [15, 22] and require extensive collaboration and information exchange. Previous research indicates the hazards associated with hospital discharge and the need of interventions to improve care transitions for people with complex care needs [15, 23–25]. The vulnerable exchange points in care transitions may increase both the need of healthcare services and healthcare costs [26]. Previous studies on care transitions highlight the importance of applying a systems thinking perspective instead of linear thinking to investigate hospital discharge as a process [27–30]. Understanding and improving care transitions is considered to be of major importance [31] and is an international healthcare priority [26, 32], as these are error-prone situations [33]. Former studies describing the discharge planning process, using the FRAM, have mapped the variability and WAD up to the discharge [27–30]. One common failure identified [30] was a lack of teamwork collaboration across hospital and community care, something the current study has focused on. One recent study [28] evaluated both pre-discharge processes, follow-up, and readmission. However, that study focused on the frail older patients’ health status between admission and potential readmission to identify challenges they faced during the transition process. The current study differs from former studies in that it incorporates multi-professional and cross-organisational team collaboration and coordination processes from all care providers involved (hospital, primary care, municipality), related to both pre- and post-discharge processes, to evaluate how discharge planning matches the needs arising when the patient returns home. This study sought to provide a detailed understanding of how coordination of care at hospital discharge is organised, including inherent interdependencies and potential system variability where avoidable adverse events may occur. Furthermore, we hoped to find how discharge planning in cross-organisational collaboration aligns with the needs that arise when the patient is enrolled in or returned to home healthcare and social care. Therefore, it was considered to be valuable to map the critical situations where alignments between demands and resources occur and where there were misalignments between how was described in procedures and intended to be done, and how it was done in practice.

The aims were to 1) map coordination and team collaboration across healthcare and social care organisations, 2) describe interdependencies and system variability in the discharge process for older people with complex care needs, and 3) evaluate the alignment between discharge planning and the needs at home.

## Methods

### Design and study setting

**Fig. 1:**
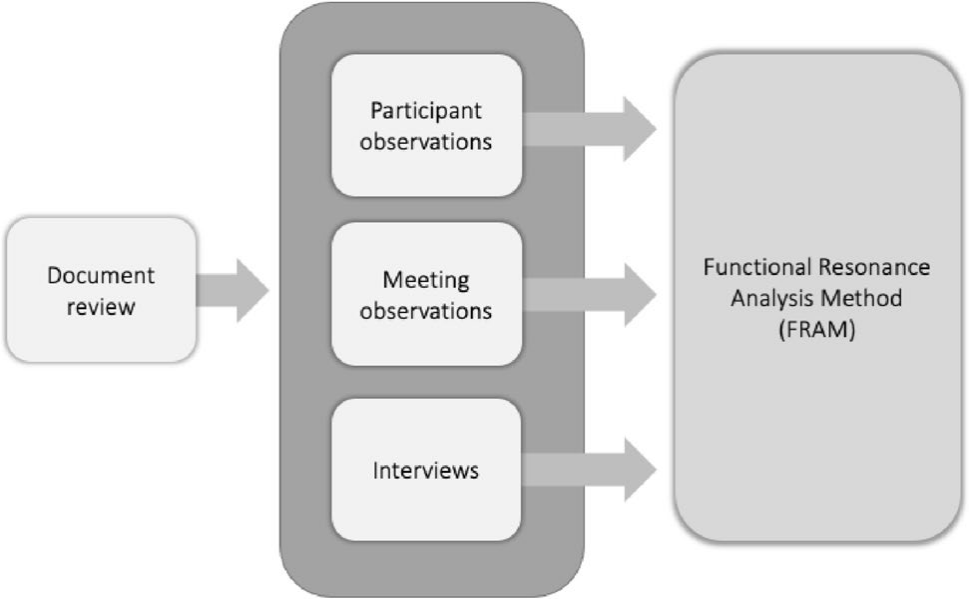
Study design with multiple convergent data collection methods

We conducted a qualitative study using multiple convergent data collection methods [34, 35] to gather data on care transitions and then analysed the data with the Functional Resonance Analysis Method (FRAM) [36] (Fig. 1). Qualitative methods comprising document review, observations and interviews with healthcare and social care professionals (HSCPs) were used for data triangulation [34]. This study focused on couplings and interdependencies in care transitions, delimited to hospital admission, in-patient care, hospital discharge and the first 72 h at home.

The study is part of a research project performed in collaboration with a region and municipalities in southern Sweden. The region encompasses three small to medium-sized hospitals, 40 primary care centres and twelve municipalities. Sweden has a decentralised healthcare system managed and run by regions and municipalities. The regions are responsible for primary, secondary and specialist care, while the municipalities provide healthcare and social care in nursing homes and home care services [37, 38].

### The Functional Resonance Analysis Method (FRAM)

The FRAM [36, 39] offers an approach to map and visualise variability and interdependencies in complex systems and examine relationships between individual processes and elements. The FRAM has been used in numerous studies within healthcare [40], providing deeper understanding on how different components interact and drive non-linear series of actions in complex healthcare processes such as transitional care [15, 28] and hospital discharge [27, 29]. In the FRAM, the discharge process can be modelled as consisting of activities that occur daily within and between organisations. The purpose of a FRAM analysis is to describe how a system should work to achieve the intended goals and to understand how potential variability can prevent this from happening or enhance functionality [36]. It is first necessary to map and construct a model of the system (as in the current study) and then instantiate the model [41] by analysing patient scenarios from WAD (to be presented in a separate study).

**Fig. 2:**
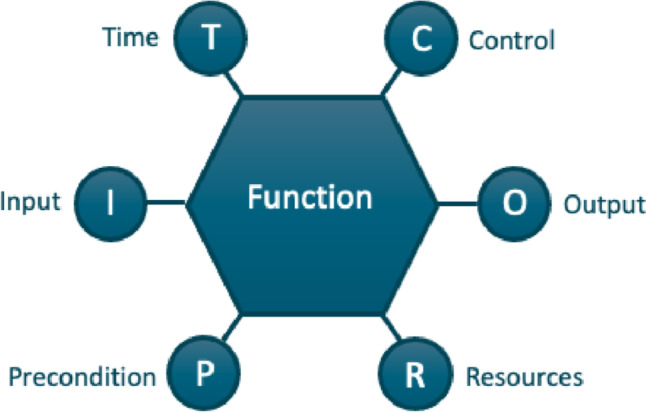
The six aspects of a function in the FRAM model [41]

In the FRAM, a function refers to the means, acts or activities that are necessary to achieve a goal or produce a certain result [36, 42]. However, a function can also refer to something the organisation does, such as ‘treat patients’, or something that a technical system does automatically or after manual input. Each function is described by six aspects – Input, Output, Precondition, Resource, Control and Time [43] – as illustrated in Fig. 2. An example of how this can be realised is presented in Table 1.

**Table 1:** Example of aspects of a function (‘Initiate planning of coordinated care’) in the FRAM model

Aspect	Description	Example
**Input**	That which starts the function. Link to previous/upstream functions What a function uses to produce the output	Need of coordinated care
**Outputs**	The result of a function. Constitutes links to subsequent/downstream functions	Initiated coordination
**Precondition**	Conditions that should be satisfied for a function to be executed	Consent from patient
**Resource**	What is needed or consumed to execute a function	Care contact
**Control**	What regulates or monitors a function	Policies for coordinated care
**Time**	Temporal constraints and requirements affecting the function	Within 24 h

The output of one function interacts or ‘couples’ with other functions. In a visual representation of a FRAM model, this is illustrated by lines or ‘couplings’ connecting the functions. Functional upstream–downstream couplings create the basis for functional resonance [41], as they mean that the variability of a function may be amplified or dampened by other system functions. Internal variability relates to the nature of a function, for example bias in decision-making or assessment or the quality and effectiveness of communication in an organisation. External variability emerges from the variability of the conditions or work environment in which the function is performed, affected by unspoken norms and expectations or by organisational factors such as regulations and legal constraints. By creating a visual of the intra- and inter-organisational complexity and variability of the care transition process, the FRAM may aid discovery of potential interdependencies, vulnerabilities and gaps where discontinuities occur, thereby potentially contributing to improved patient safety.

### Sampling and participants

A purposive sampling strategy was adopted [44] to recruit participants from various professions and care providers involved in care transitions from hospital to home, to maximise data variation [44]. The extensive sampling enabled capture of the intra- and inter-organisational perspectives of stakeholders involved in the delivery of healthcare and social care in such transitions. The operation managers from each organisation received an introduction to the project through written and verbal information. They in turn informed their personnel. All participants were informed prior to observations and interviews and gave informed consent to participate in the study.

The sample consisted of 60 HSCPs from in-hospital care, ambulance care, primary care and municipal care. The participants had a range of professions and roles: registered nurses, physicians, care coordinators, occupational therapists, physiotherapists, social service officers, assistant nurses, ambulance nurses, as well as first-line managers. This provided a multidisciplinary and cross-stakeholder perspective on collaboration in care transitions.

### Data collection and procedure

The data collection was performed from June 2020 to October 2021, as an iterative and reflexive process, converging data from observations, interviews, and document review. Our use of multiple data sources allowed for convergence, to achieve comprehensive data and to expand and enrich the understanding of the complex care transition processes [34]. The data collection encompassed a total of 45 interviews, eleven participant observations and five meeting observations (Table 2).

**Table 2:** Data collection and participants

**2a)**				
**Data collection**	**n**			
Interviews	45			
Participant observations	11			
Meeting observations:	5			
Coordination meeting	2			
Care planning meeting	2			
Discharge conversation	1			
**2b)**				
			**Occurrences in**	
**Participants**	**n** (60)	**Interviews** (45)	**Participant observations** (11)	**Meeting observations** (24)
Medical and geriatric hospital wards	(12)	(7)	(6)	(12)
Registered nurse	5	2	4	3
Care coordinator	4	4	2	4
Physician	3	2		1
Ambulance care	(5)	(5)		
Ambulance nurse	5	5		
Primary care	(8)	(3)	(2)	(2)
Care coordinator	6	3	2	4
Physician	2	2		
Municipal care	(35)	(27)	(3)	(14)
Occupational therapist	5	4		2
Physiotherapist	6	4		3
Registered nurse	8	7		2
Social service officer	5	2		4
Care coordinator	5	4	3	3
Assistant nurse	4	4		
First-line manager	2	2		

#### Document review

First, to develop observation protocols and questions for interviews, written materials were collected by searching and accessing available documents on government websites as well as digital and printed versions used in everyday work at the units being observed. Documents included in the review were national laws [45–48], regional guidelines (*n* = 3) and local routines from in-hospital care (*n* = 2) and municipal care (*n* = 2) describing the discharge process. The documents were analysed using document review, mapping the discharge process according to regulations and routines (i.e., WAI [41]).

#### Observations

To gain an understanding of how the everyday work was carried out [36], participant observations were performed [35] in multiple contexts. The researcher (nurse background) was present and recognisable to the participants, but not an active participant with a role in the social context, enabling observation and occasional interactions leading to a high level of involvement while maintaining detachment [35]. Before meeting observations, all participants were informed and gave consent. To protect the privacy of all parties, as well as to avoid gathering sensitive information like patient data, none of the meetings were recorded. Instead, the observer wrote fieldnotes, in line with participant observation methods [35].

A semi-structured observation guide was used, building on the understanding of WAI gained from the document review. It encompassed themes relevant to care transitions and hospital discharge, such as coordination, communication, and information exchange. Observations were complemented with contextual inquiries [35] to understand what was happening (for example ‘Could you tell me about what you are doing there?’). These observations were performed in hospital wards (*n* = 6), primary care (*n* = 2) and community settings (*n* = 3) for a total of 86 h, with personnel shadowed in their daily work. Various forms of meetings were observed, including coordination meetings, care planning meetings and discharge conversations between patients and physicians. Fieldnotes taken in association with the observations were rewritten later the same day.

#### Interviews

The knowledge gleaned from the document review was expanded through interviews with first-line managers. These interviews related to the work performed at the management level and what tasks were supposed to be performed in the discharge process, contributing to the understanding of WAI. The interviews were recorded and transcribed verbatim.

To create a more in-depth view of the research problem, and to study different ways of understanding the work processes in care transitions, the observations were complemented with semi-structured interviews with personnel from throughout the care trajectory. The interviews were conducted on site, via telephone or via Skype/Zoom and lasted 32–75 min (mean 57.8, SD 21.2). An interview guide was developed and revised during the process, reflecting the analytical process and addressing the gaps in understanding remaining after the observations. The interviews thus offered a deeper, clarifying perspective on actions already observed. The interviews were recorded and then transcribed verbatim.

### Data analysis

First, materials from the document review and interviews with managers were analysed using manifest content analysis [49], mapping WAI. The materials were coded and then combined by comparing and contrasting them, in order to further abstract the codes into categories describing the different steps in the discharge process [50].

The four-step approach described by Hollnagel [41] was applied for the analysis of WAD, preceded by a preparation step (Step 0), as follows:

- Step 0: Define the purpose of the FRAM analysis.
- Step 1: Identify vital functions that are required for everyday work to succeed.
- Step 2: Determine and describe the system’s potential for variability.
- Step 3: Identify functional dependencies and potential for functional resonance that may affect the system.
- Step 4: Propose ways to manage possible occurrences of uncontrolled performance variability.

Data analysis and creation of the FRAM model of WAD were performed through an iterative and reflexive process of identifying patterns in the transcribed data and fieldnotes from observations, to find a foundation in which existing work and communication processes could be recognised as functions. The datasets were combined by comparing and contrasting the data, with contextual factors investigated and taken into consideration. Differences and similarities in the process description were identified and discussed by the researchers to allow emerging contradictions to promote a deeper understanding and breadth of perspectives from the participants, which further illuminated the complexity of the phenomenon.

The functions identified were then listed in a Microsoft Excel spreadsheet. For each function, a detailed description including as many aspects (time, control, input, output, resources, and preconditions) as possible was identified and described, to create an understanding of how the process could be performed. Then, a preliminary chronology was drafted to position the functions in order based on their internal couplings. The functions were revised until final consensus was reached.

Next, functions that – at an abstract level – made up the discharge process were identified. These functions were stepwise extended into a comprehensive model of everyday work in care transitions from hospital to home, visualised using the ‘FRAM Model Visualizer’ [42]. The model was validated through expert audits by managers and HSCPs involved in discharge process [15] with recognition and affirmation of the process, confirming the relevance of the results and the management of contradictory views. Hence, no revision of the model was needed. Then, the potential variability of function output was assessed [41] based on what can reasonably be expected to happen in the clinical setting, based on observational and interview data. The variability determined the quality of the output, which in turn influenced aspects of other functions in the system. The FRAM model was thus used to understand how variability and subsequent adjustments can affect other functions and thus the discharge process as a whole. Based on different phenotypes such as timing, precision, and duration, previously suggested as variability manifestations [41], two analytical clusters (timing and precision) emerged in this process. The clusters characterised how the interdependence of functions could potentially lead to unexpected results, depending on how the outputs of each function could vary in timing (too early, on time, too late, not at all) and precision (precise, acceptable, imprecise), from the perspective of the needs of downstream functions. This step was followed by identification of functional dependencies and potential for functional resonance that could affect the system’s ability to perform the discharge process successfully. The couplings made it possible to analyse and describe how variability of the output from one function could affect other functions, without any claims of a cause-effect perspective. Lastly, the couplings and dependencies between functions informed proposals on ways to manage possible occurrences of uncontrolled performance emerging from implementation of changes that either dampened negative effects and absorbed variability or reinforced positive effects and amplified variability [51].

## Results

The results of this study are presented in five subsections. The first subsection relates to the discharge process as described in regulations and policy documents (WAI), while the three subsequent subsections present WAD, using the FRAM steps to outline potential gaps within the care transition process. Based on the results of the analysis (steps 1–3), measures to manage variability, risks, and gaps are suggested in the final step.

### The discharge process as described in regulations and policy documents

The review of laws, regulations, and policy documents showed that the discharge process was intended to be achieved through eight overarching steps involving multiple stakeholders from several professions, as well as the patient and family. The steps, listed in Table 3, represent WAI and serve to achieve the goal of safe and secure discharge from in-patient care.

**Table 3:** The eight steps describing the hospital discharge process as work-as-imagined

Step in process	Description of action
**Identify**	Identify patients in need of coordinated care
**Initiate**	Initiate coordination process
**Plan**	Plan and prepare for discharge
**Agree**	Jointly determine and agree that the patient is fit for discharge
**Facilitate**	Facilitate the care transition from hospital by supplying medicines, lists and care aids
**Transfer**	Transfer information and care responsibility from one care provider to another
**Implement**	Implement and perform the coordinated efforts in the home
**Re-evaluate**	Re-evaluate and reconsider the coordinated efforts in light of perceived needs after initiating the planned efforts

The final step of re-evaluating also includes identification of increased or altered needs for coordination, which may lead to the beginning of a new cycle of care transition.

### Vital functions required for everyday work to succeed

Hospital discharge as performed in practice was modelled as a complex multi-stakeholder process (Table 4), describing relevant coordination and activities that occurred daily within and between different parts of the organisations, constituting WAD.

**Table 4:** Work-as-done in the hospital discharge process as modelled by the FRAM

Function (i.e., event)	Description	
Admit patient to ward	A patient is assessed as needing inpatient care. If there are hospital beds available, the patient is admitted to the ward and treated and cared for by healthcare professionals	
	**Identified aspects:**	
	**Input**	Patient to hospital
	**Output**	Patient to inpatient care
		During inpatient care
	**Precondition**	Need of inpatient care
	**Resource**	Availability to beds on ward
	**Control**	Decision on admission
		Decision to re-admit patient
Adapt aids at home	When there is an altered need of aids, the aids supplied may be altered, depending on availability	
	**Identified aspects:**	
	**Input**	Altered need of aids
	**Output**	Adequate aids in home
Adapt home care	When medical or care needs are altered, the home care efforts may be adapted to better suit the patient's needs	
	**Identified aspects:**	
	**Input**	Altered need of home care
	**Output**	Adequate home care efforts
Adapt social care at home	When there is an altered need of social care, the social care efforts may be altered to better suit the patient's needs	
	**Identified aspects:**	
	**Input**	Altered need of social care
	**Output**	Adequate social care efforts in home
Assess ADL and mobility	If the patient needs coordinated care after discharge, an assessment of ADL and mobility is conducted to get an understanding of what support and aids the patient may need after discharge. This is done by rehab personnel such as physical therapists or occupational therapists at hospital, depending on availability. This assessment is otherwise performed by nurses on the ward	
	**Identified aspects:**	
	**Input**	Initiated care coordination process
	**Output**	Rehab assessment
	**Resource**	Availability to rehab personnel
Assess patient in prehospital setting	If there is a perceived need of prehospital assessments and care, the municipal staff, family or patient may call emergency services. The ambulance personnel then conduct an assessment in the prehospital setting	
	**Identified aspects:**	
	**Input**	Perceived need of prehospital assessment and care
	**Output**	Need of inpatient care
		Emerging needs
		Inadequate possibility of care in home
	**Resource**	Adequate care efforts in home
	**Control**	Individual plan of care
		Medical plan at exacerbation
		Medical assessment or dialogue
Assess patient ready for discharge	When the medical treatment is completed, the patient is assessed as medically fit for discharge and the physician can assess whether the patient is ready for discharge. The next step is then to discharge the patient so they can go home. If there is a need for coordinated care and the patient needs new or altered care efforts at home, the physician may delay discharge until preparations for the efforts are completed	
	**Identified aspects:**	
	**Input**	Medically treated patient
	**Output**	Patient ready for discharge
		As soon as possible
		Before discharge
Assign care contact	Primary care designates a person as a care contact for the patient. This person can call for a CIP (Coordinated Individual Planning)	
	**Identified aspects:**	
	**Output**	Care contact
Call for a CIP in home	The permanent care contact in primary care calls for a CIP at home no later than three days after the discharge notice	
	**Identified aspects:**	
	**Input**	Need for CIP in home
	**Output**	Call for CIP
	**Precondition**	Patient ready for discharge
	**Resource**	Care contact
	**Control**	Plan for evaluation
	**Time**	Within 3 days
Check bed availability	The physician checks if there are beds available on the ward in question. If the patient has a great need of inpatient care and there are no beds available, they might be placed on a different ward	
	**Identified aspects:**	
	**Output**	Availability to beds on ward
Co-create plan of coordinated care	There is an ongoing dialogue between the various units involved in the patient’s case. Regular status updates are communicated from healthcare professionals in inpatient care. Questions are asked between the units to plan and coordinate efforts and aids before the patient returns home. A preliminary plan for the care efforts is written after dialogue between all the parties involved	
	**Identified aspects:**	
	**Input**	Initiated care coordination process
	**Output**	Prepared plan for care in home
		Plan for evaluation
		Plan for aid tools in home
		Possibility of dialogue
	**Precondition**	Consent from patient
		Rehab assessment
		Need of coordinated care
	**Resource**	Knowledge of patient
		Knowledge of family
		Interprofessional collaboration
		Digital communication for collaboration
	**Control**	Needs of patient
		Needs of family
	**Time**	During inpatient care
Collaborate within and between organizations	A lot of planning and dialogue takes place within and between the various healthcare organizations, though the patient and family do not appear to be represented	
	**Identified aspects:**	
	**Output**	Interprofessional collaboration
Conduct admission conversation with patient	When admitting a patient, the physician and the registered nurse on the ward conduct an admission conversation with the patient to acquire knowledge of their situation, symptoms and needs. At this juncture, family can also be involved or contacted. The patient's requests for care are listened to. Patients and family are offered the chance to share their views on assumed needs when returning home and whether there is a need for coordination of care	
	**Identified aspects:**	
	**Input**	Patient in inpatient care
	**Output**	Requests from patient or family
		Needs of patient
Consult physician in primary care	When there is a need for medical assessment of the patient, for example in case of exacerbation of a chronic illness, a dialogue with the primary care physician responsible for patient care is requested	
	**Identified aspects:**	
	**Input**	Need of medical assessment or dialogue
	**Output**	Decision to re-admit patient
		Medical assessment or dialogue
	**Resource**	Individual plan of care
Decide on admission	The physician decides whether to admit the patient to the ward	
	**Identified aspects:**	
	**Output**	Decision on admission
	**Precondition**	Need of inpatient care
	**Resource**	Availability to beds on ward
Decide upon level of care	An increased need of medical care leads to a decision about what may be the most appropriate level of care. To inform this decision, the medical plan and agreed plan for care at home are used. The patient should preferably receive adapted care efforts at home, but if this is not possible, an escalation of care may be needed, where the patient is admitted to hospital	
	**Identified aspects:**	
	**Input**	Increased need of medical care
	**Output**	Inadequate possibility of care in home
		Need of medical assessment or dialogue
		Perceived need of prehospital assessment and care
	**Resource**	Adequate care efforts in home
	**Control**	Medical plan at exacerbation
		Decision on medical care in home
		Agreed upon plan for care in home
		Individual plan of care
Discharge patient	The attending physician discharges the patient from inpatient care, thus terminating their inpatient trajectory in this case	
	**Identified aspects:**	
	**Input**	Patient ready for discharge
	**Output**	Patient discharged
		Continuous medical plan in primary care
		Discharge letter to patient
		Updated list of medicines
	**Resource**	Patient ready to go home
	**Control**	Agreed upon time for homecoming
Enclose medicines at discharge	New medicines that have been added to the medication list are enclosed for the first few days at home. This is to ensure that the patient gets the medicines even if the pharmacy is closed, for example during discharge on the weekend	
	**Identified aspects:**	
	**Input**	Patient goes home
	**Output**	Updated list of medicines
Escalate care	If the situation at home is untenable despite care efforts or when the patient needs inpatient care, the patient is readmitted to hospital	
	**Identified aspects:**	
	**Input**	Inadequate possibility of care in home
	**Output**	Decision to re-admit patient
		Patient to hospital
Get consent to share information	Healthcare professionals contact the patient (or family if the patient does not have the ability to consent) to get consent to share information across caregiver boundaries. Without such consent, the coordination process cannot proceed	
	**Identified aspects:**	
	**Output**	Consent from patient
Handle needs with available resources	When new or altered needs emerge, they may be handled by family or through self-care, if their abilities are sufficient to achieve adequate care	
	**Identified aspects:**	
	**Input**	Emerging needs
	**Output**	Adequate care efforts in home
	**Resource**	Support from family
		Self-care
Have capacity for care and social support	The capacity and ability of the family to provide or aid in care and social support of the patient	
	**Identified aspects:**	
	**Output**	Ability to provide care and social support
Have capacity for self-care	The patient’s knowledge, experience and ability for self-care	
	**Identified aspects:**	
	**Output**	Ability in self-care
Identify altered care needs	During the continued care at home, the patient, family and healthcare professionals pay attention to any changes that may indicate altered care needs and act accordingly	
	**Identified aspects:**	
	**Input**	Monitoring of care efforts
		Attention to patient health status
		Assessment of efforts in home according to plan
	**Output**	Altered need of social care
		Altered need of home care
		Altered need of aid tools
		Emerging needs
		Increased need of medical care
Identify need of coordinated care	An admission conversation is conducted with the patient where the physician or other healthcare professional determine the need for coordinated care after returning home. Admission to hospital may have been preceded by a referral from primary care which may clearly state that there is a need for coordinated care. Alternatively, the patient may have been assessed by a registered nurse in home care before hospitalization, who has made clear that there is a need for coordinated care. The patient or family can also communicate their wishes or needs for coordination of care to the healthcare professionals. A prerequisite to considering coordination of care is that the patient consents to information sharing between healthcare providers	
	**Identified aspects:**	
	**Input**	Patient in inpatient care
	**Output**	Need of coordinated care
		Within 24 h
	**Precondition**	Consent from patient
	**Resource**	Requests from patient or family
		Referral from municipal or primary care
		Knowledge of patient
		Knowledge of family
	**Control**	Needs of patient
		Needs of family
Inform family of discharge	No later than the day the patient is returning home, the family of the patient is contacted by the ward and informed of this	
	**Identified aspects:**	
	**Input**	Patient ready to go home
	**Output**	Family ready to receive patient at home
		Agreed upon time for homecoming
	**Time**	Before discharge
Initiate planning of coordinated care	Inpatient healthcare professionals make an admission announcement in the digital system Cosmic Link, where the estimated date of discharge is presented. Further, healthcare professionals assess and announce the need for efforts and describe the status of the patient. A coordination case is created and units with ongoing efforts for the patient are linked to the case	
	**Identified aspects:**	
	**Input**	Need of coordinated care
	**Output**	Initiated care coordination process
	**Precondition**	Consent from patient
	**Resource**	Care contact
	**Control**	Policies for coordinated care
	**Time**	Within 24 h
Install aids at home	Rehab personnel meet with the patient upon their return home. The staff test and install the necessary aids. Some aids and adaptations may take longer than others to implement and therefore the patient may be at home for several days before they are used	
	**Identified aspects:**	
	**Input**	Patient goes home
	**Output**	Aid tools in home
	**Precondition**	Rehab assessment
		Aid tools available
	**Control**	Plan for aid tools in home
	**Time**	Patient goes home
Invite patient and family in dialogue	When care efforts for the return home are planned, patients and family are invited to participate in this dialogue, for instance in discharge planning meetings	
	**Identified aspects:**	
	**Output**	Needs of family
		Needs of patient
		Knowledge of patient
		Knowledge of family
Monitor and evaluate care efforts at home	An evaluation of the planned and performed care efforts is made in the patient’s home within a couple of days, together with the patient, family and relevant healthcare professionals. Based on what has worked well and less well, adjustments can be made	
	**Identified aspects:**	
	**Input**	Patient goes home
	**Output**	Monitoring of care efforts
		Assessment of efforts in home according to plan
	**Precondition**	Execution of efforts in home according to plan
	**Resources**	Call for CIP
	**Control**	Individual plan of care
		Plan for evaluation
	**Time**	Days after homecoming
Notify receiving units of pending discharge	As soon as possible after the decision has been made that the patient is ready for discharge, the receiving units are notified of this, so they can start preparing to receive the patient	
	**Identified aspects:**	
	**Input**	Patient ready for discharge
	**Output**	Units informed of discharge
		Within 3 days
		Need for CIP in home
	**Time**	As soon as possible
Perform care transition	The care transition from hospital to home takes place in connection with the patient being discharged by the attending physician. Healthcare professionals on the ward ensure that the patient comes home to their accommodations and any transport needed is ordered	
	**Identified aspects:**	
	**Input**	Patient discharged
	**Output**	Patient goes home
		Days after homecoming
	**Precondition**	Patient ready to go home
		Municipal efforts ready
		Family ready to receive patient at home
		Updated list of medicines
		Medicines for homecoming
		Prepared municipal home care efforts
	**Resource**	Information from inpatient care
		Discharge letter to patient
Perform CIP	A coordinated individual plan is made, usually within the first few days of homecoming. Several professionals collaborate in this meeting together with patient and family, to make sure the care efforts and aids are appropriate for the current needs	
	**Identified aspects:**	
	**Input**	Call for CIP
	**Output**	Individual plan of care
	**Time**	Days after homecoming
Perform coordinated care at home	Self-care and care efforts are carried out at home by patients, family and social care along with registered nurses from the municipality. The intended care is based on the medical plan from primary care and the agreed plan devised during the care planning before discharge. When returning home from hospital, the patient receives information both verbally and in writing from the hospital regarding their care and treatment, such as medicine changes	
	**Identified aspects:**	
	**Input**	Patient goes home
	**Output**	Execution of efforts in home according to plan
		Attention to patient health status
	**Precondition**	Medical plan and treatment in primary care
		Adequate aid tools in home
		Adequate social care efforts in home
		Adequate home care efforts
		Consent from patient
		Updated list of medicines
		Medicines for homecoming
		Adequate care efforts in home
		Patient met in home
	**Resource**	Information from inpatient care
		Booked social care personnel
		Aid tools in home
		Summary of medical care
		Care contact
		Self-care
		Support from family
	**Control**	Agreed upon plan for care in home
		Decision on medical care in home
Perform discharge planning	Before the patient is discharged, a discharge planning is performed. This is often performed as a hybrid meeting where the patient (and if possible, a member of the family) and the nurse from the ward or a designated care coordinator attend physically at the hospital, while professionals from primary care and municipal care attend via video. At this meeting, the patient is informed of the prepared plan for care in home and this is agreed upon in dialogue with the family	
	**Identified aspects:**	
	**Input**	Patient ready for discharge
	**Output**	Informed patient and family
		Agreed upon plan for care in home
	**Precondition**	Consent from patient
	**Resource**	Possibility of dialogue
		Interprofessional collaboration
		Digital communication for collaboration
		Care contact
	**Control**	Knowledge of family
		Knowledge of patient
		Prepared plan for care in home
		Policies for coordinated care
	**Time**	Before discharge
Perform self-care	The patient performs self-care at home depending on their own ability and physical condition. The self-care is guided by the information from inpatient care	
	**Identified aspects:**	
	**Output**	Self-care
	**Resource**	Ability in self-care
	**Control**	Information from inpatient care
Plan and order aids	Rehab personnel plan what aids may be needed at home after discharge. This depends on the assessment of the patient’s ADL and mobility. The rehab personnel order the aids needed and make sure they are available when the patient returns home	
	**Identified aspects:**	
	**Input**	Patient ready for discharge
	**Output**	Aid tools available
	**Precondition**	Rehab assessment
		Agreed upon plan for care in home
Plan and schedule social care personnel	When a decision is made regarding when it is believed the patient will be discharged and go home, the care coordinator may plan for home visits and schedule this for the social care personnel. The social care personnel are then also informed that the patient is on their way home and what interventions will be needed. The earlier this is done, the greater the chances of bringing in enough staff to perform all these tasks while still having time for other assignments already planned	
	**Identified aspects:**	
	**Input**	Units informed of discharge
	**Output**	Booked social care personnel
		Informed social care personnel
	**Precondition**	Agreed upon plan for care in home
Plan municipal home care	The municipal home care nurses plan for what will be needed when the patient returns home. Examples could include wound dressing or distribution and administration of medicines	
	**Identified aspects:**	
	**Input**	Patient ready for discharge
	**Output**	Prepared municipal home care efforts
	**Precondition**	Agreed upon plan for care in home
Prepare care transition to home	Care units, patients and families are prepared for the discharge and the patient to return home. A discharge planning meeting is held where the patient and family talk to healthcare professionals in the care units that are discharging and receiving the patient at home. Aids are ordered and planned to be installed and provided in the patient's home upon their return home. Social care personnel are notified that the patient is coming home, what time they should meet them and what measures are to be taken. Registered nurses in home care set up care plans and medical records for the patient and plan visits, medicine administration and home care efforts for when the patient comes home	
	**Identified aspects:**	
	**Input**	Patient ready for discharge
		Units informed of discharge
	**Output**	Patient ready to go home
		Latest the same day as discharge
		Municipal efforts ready
		Before discharge
		Medicines for homecoming
	**Precondition**	Consent from patient
		Informed patient and family
	**Resource**	Plan for aid tools in home
		Informed social care personnel
	**Control**	Agreed upon plan for care in home
Receive patient in home	Home care personnel or rehab personnel meet the patient upon their arrival at home after discharge. This requires that the municipal efforts are ready and that social care personnel are both scheduled and informed about the patient’s status and needs	
	**Identified aspects:**	
	**Input**	Patient goes home
	**Output**	Patient received in home
	**Precondition**	Municipal efforts ready
		Booked social care personnel
		Informed social care personnel
	**Time**	Agreed upon time for homecoming
Request rehab resources	Rehab personnel are available to perform an assessment of ADL and mobility	
	**Identified aspects:**	
	**Output**	Availability to rehab personnel
Resume medical responsibility in primary care	The physician in primary care receives a referral from the treating physician in inpatient care and thereby gets a medical assignment for follow-up or further medical measures to be performed after inpatient care. This can also determine parts of the care performed at home	
	**Identified aspects:**	
	**Input**	Referral to primary care
	**Output**	Medical plan and treatment in primary care
		Decision on medical care in home
	**Precondition**	Summary of medical care
	**Resource**	Medical plan at exacerbation
Send referral to primary care	A referral is sent to co convey the medical plan and transfer responsibility for ongoing care to primary care	
	**Identified aspects:**	
	**Input**	Continuous medical plan in primary care
	**Output**	Referral to primary care
Support care at home	In addition to the self-care performed by the patient, the family may also support care at home, depending on their abilities. This support can be used as a dampening factor if care is not functioning or coordinated, with family stepping in instead	
	**Identified aspects:**	
	**Output**	Support from family
	**Precondition**	Information from inpatient care
	**Resource**	Ability to provide care and social support
Take part of information from other caregivers	Physicians at the hospital can contact primary care or read medical records to get information, see plans, etc. about the patient's long-term care. Admission to a hospital can also be initiated after assessment and a referral by a registered nurse in home care or a physician in primary care	
	**Identified aspects:**	
	**Output**	Referral from municipal or primary care
Transfer information from inpatient care	Information is transferred between inpatient and primary care, municipal health and social care, and to patients and family. The information is provided both verbally and in writing	
	**Identified aspects:**	
	**Input**	Patient ready to go home
	**Output**	Information from inpatient care
	**Time**	Latest the same day as discharge
Treat patient in hospital	The medically responsible physician on the ward treats the patient. It is possible to review documentation and medical records from the patient's previous and ongoing care and treatment in primary care. The physician at the hospital can also contact the physician responsible for the patient in primary care, to discuss the focus of care, treatment and plans	
	**Identified aspects:**	
	**Output**	Medically treated patient
	**Precondition**	Patient in inpatient care
	**Resource**	Documentation from primary care
Update list of medicines	At discharge, the current list of medicines needs to be updated by the attending physician. This list is then printed and handed to the patient to take home. The list is also made available digitally, to be accessed by primary care and municipal home care nurses	
	**Identified aspects:**	
	**Input**	Patient ready for discharge
	**Output**	Updated list of medicines
Use digital systems for collaboration	Digital systems are used for healthcare and social care professionals to communicate in writing and via video meetings to update each other on the status of the patient and the presumed needs at home	
	**Identified aspects:**	
	**Output**	Digital communication for collaboration
Use policies on coordination of care	There are explicit guidelines and laws regarding discharge from inpatient care and what steps should be performed and by whom. There are also criteria regarding when, where and how coordination should take place	
	**Identified aspects:**	
	**Output**	Policies for coordinated care
Write medical summary	When the medical care is concluded in inpatient care, the physician writes a medical summary that is made available digitally, but is also printed and handed to the patient to take home	
	**Identified aspects:**	
	**Input**	Patient discharged
	**Output**	Summary of medical care
		Medical plan at exacerbation

**Fig. 3:**
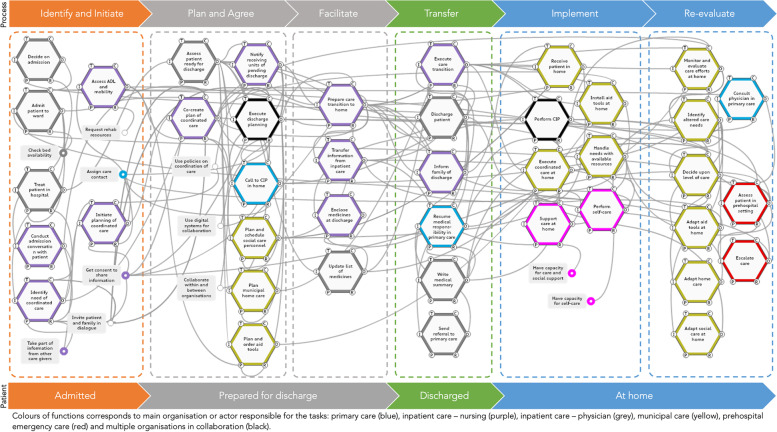
Work-as-done in relation to the discharge process and the status of the patient

The comprehensive model encompassed a sizeable number of functions (*n* = 52) required for the discharge process to succeed. In the visual presentation (Fig. 3), the functions have been clustered to follow the patient and their location through the different phases that constitute the discharge process (below the FRAM model). Additionally, WAD is illustrated in relation to the phases of the transition process in WAI (above the FRAM model), which is described by eight steps (Table 3). Furthermore, different colours show the range of actors involved in each function.

The model begins with the function ‘Admit the patient to the ward’ located in the ‘Identify and Initiate’ phase, as this function indicates the start of an in-hospital stay subsequently leading to a hospital discharge. During this initial phase, the patient is treated at the hospital ward and the potential need of coordinated care after discharge is assessed and identified, constituting the function ‘Identify the need of coordinated care’. If appropriate and the patient consents, the care coordination process is initiated by the function ‘Initiate planning of coordinated care’. During the ‘Plan and Agree’ phase, the future needs of home care, social support, and care aids are planned for and agreed upon, leading to the function ‘Co-create plan of coordinated care’. A coordinated individual planning meeting is performed (‘Execute discharge planning’) or scheduled to be held at the patient’s home after discharge. Next, the care transition is facilitated by transferring information between stakeholders (‘Transfer information from in-hospital care’) and the list of medicines is updated. To facilitate for home care personnel to administer new or changed medicines to the patient in the first days after their return home, the medicines in question are sent along with the patient by the hospital nurse. The next phase is ‘Transfer’, where the patient is discharged and returns home. Here, the function ‘Execute care transition’ marks the point in the process where a transition in both the physical location and caregiver responsibility is performed. There is thus a transfer of responsibility where primary care (re-)assumes medical responsibility for the patient, guided by any referrals from the treating physician at hospital. During the ‘Implementation’ phase, the patient is received at home, initiating ‘Execute coordinated care’ where care aids are installed and care needs are handled with available resources. After the return home, during the ‘Re-evaluate’ phase, coordinated care is re-evaluated to consider new or altered care needs or exacerbations in pre-existing illnesses. If possible, actions may be taken to respond to the altered needs with available resources through the functions ‘Adapt home care’, ‘Adapt social care’, or ‘Adapt care aids’. If this adaptation is insufficient in relation to the patient’s needs, the physician in primary care may be consulted or an ambulance called to provide prehospital assessment, care, and transport back to hospital if required.

### Potential variability in the system

The model of the discharge process reveals normal everyday changeability in the functional output, reflecting potential variability. Variability with the potential to cause impacts on other parts of the process is most apparent in functions related to the planning and preparation for the care transition (during the ‘Plan and Agree’ and ‘Facilitate’ phases) and the evaluation at home (the ‘Re-evaluate’ phase) based on timing and precision of performance. The potential for internal variability relates to bias in assessments of what care aids a patient may need on arrival at home (originating from the functions ‘Assess ADL and mobility’ and ‘Plan and order care aids’), or whether a patient is truly ready for discharge (‘Assess patient ready for discharge’). The quality of communication via digital systems can also be a factor complicating the assessment of care needs. External variability is exemplified by the legal constraints limiting the transfer and sharing of patient information between the stakeholders involved in a care transition or by the limited personnel resources available when a patient is discharged late in the afternoon. Examples of potential variability related to timing and precision in a selection of functions are presented in Table 5.

**Table 5:** Examples of potential for variability in the hospital discharge process

Function	Output	Potential for variability		Expressions of variability
		**Timing**	**Precision**	
*Initiate planning of coordinated care*	Initiated care coordination process	Too early		Input possibly missed, risk of lacking information for correct decision
		On time		No effect or possible damping of further variability
		Too late / Omission		Delayed or non-initiation of coordination may mean delaying the case or other healthcare providers being given a very short time to communicate and plan
			Precise	Possible damping of further variability
			Acceptable	No effect
			Imprecise	Misjudging the need for coordinated care (the patient is excluded from the subsequent process and discharged without intervention)
*Co-create plan of coordinated care*	Agreed upon plan for care at home	Too early		A care plan created too early in the patient’s hospital care risk becoming imprecise due to the dynamic conditions of recovery. Depending on the status of the patient, the care plan may need to be re-created
		On time		Possible damping of further variability
		Too late / omission		Delayed or non-initiation of coordinated care plan may mean delaying the case or other healthcare providers being given a very short time to communicate and plan
			Precise	Possible damping of variability as a precise care plan will aid in situations that may arise downstream
			Acceptable	No effect or increased variability due to ambiguity
			Imprecise	Incorrect depiction of current care efforts and aid tools at home may misrepresent the support available to the patient when discharged
*Prepare care transition to home*	Patient ready to go home	Too early		Increased variability as the patient may not be ready for discharge or the preparations made will not be relevant, the function may need to be repeated
		On time		Possible damping of further variability
		Too late / omission		Ad hoc solutions are used to prepare the patient with limited time
			Precise	Possible damping of variability
			Acceptable	Increases variability downstream to compensate for flaws in preparation
			Imprecise	In lack of accurate preparation, the correct medications, adapted aid tools or care efforts will not be available when the patient comes home. The municipal staff will then need to devote considerable time to try to solve this, taking time from other patients and assignments
*Execute care transition*	Patient goes home	Too early		The discharge of a patient frees up a hospital bed, making it available to a new patient. However, when a patient is discharged prematurely, the patient may not actually be ready for discharge increasing the workload for the home care personnel with the risk of readmissions if the situation becomes untenable in the home
		On time		Possible damping of variability
		Too late		A discharge late in the afternoon presses the home care personnel to receive the patient with less personnel on duty and limited availability to contact managers in need of added resources
		Omission		May contribute to resource issues in the hospital ward if the patient could have been discharged successfully but is now occupying a hospital bed
			Precise	Possible damping of variability
			Acceptable	No effect
			Imprecise	Increased variability in downstream functions to compensate for missed aspects
*Execute coordinated care at home*	Execution of efforts in home according to plan	Too early		-
		On time		Dampening of variability
		Too late		Delayed activity affecting the care of the patient, ad hoc solutions
		Omission		Ad hoc solutions or potential omitted care of the patient
			Precise	Possible dampening of variability
			Acceptable	No effect
			Imprecise	If the preparation of care efforts does not correspond to the actual needs, it may mean a lack of care and social support for the patient and "firefighting" by the various actors to solve this
*Monitor and evaluate care efforts at home*	Assessment of efforts in home according to plan	Too early		If the follow-up occurs too early, the patient has not had time to settle at home or to know what works and what does not
		On time		Dampening of variability, promoting a safe and secure care adapted to the patient’s care needs
		Too late / Omission		If it takes too long or the evaluation is omitted, there may emerge urgent needs or problems due to the care measures not being adapted to the current care needs and resources
			Precise	Dampening of variability
			Acceptable	No effect
			Imprecise	Increased variability
*Handle needs with available resources*	Adequate care efforts in home	Too early		Increased variability, possibility of missing actual care needs
		On time		Dampening of variability, promoting a safe and secure care adapted to the patient’s care needs
		Too late		Increased variability
		Omission		Increased variability
			Precise	Dampening of variability
			Acceptable	No effect
			Imprecise	Timing of and variability in upstream functions determines the availability of resources for the patient. Depending on the day of the week and the time of day, the resulting needs can be handled either at short notice, with a large amount of effort or not at all. May result in a readmission in hospital when all options are exhausted at home

### Functional dependencies and potential for functional resonance

The complexity of the system becomes apparent as functional interdependencies that embody the multidisciplinary teamwork in care transitions. For example, the variability in the function ‘Execute care transition’ as performed by hospital personnel can affect the circumstances for municipal personnel to execute downstream functions, creating the basis for functional resonance.

One of the functions with the most couplings upstream and downstream is ‘Execute coordinated care at home’. The quality of the output is affected by numerous upstream functions that will amplify or dampen the variability, depending on the timing and precision of their performance. Consequently, when healthcare personnel perform coordinated care in a patient’s home, the quality of the outcome depends on the previous performance of functions in the planning and preparation for hospital discharge and care transition. When necessary, adjustments are made by municipal personnel to get the work done. If the care needs of a patient are more extensive than the assessment and planning had prepared for, the personnel will be unable to follow protocol. Instead, they must resort to using ad hoc solutions or workarounds to get the work done, which may trigger functional resonance, as the variability in output of the functions creates a suboptimal basis for downstream functions.

The functional dependencies and potential for functional resonance are here clustered into the perspectives of timing and precision.

#### Timing

Timing is essential within the WAD process as it may impact on both the prerequisites for and the outcomes of a care transition. If a patient is discharged from hospital while still in recovery, the course of the next few days will be dynamic. If care planning is performed too early, the patient’s status may change and no longer match the decisions made, resulting in inadequate care or a need to review these decisions:

*It all depends on when in time you have the discharge or care planning. Sometimes it happens quickly and then things happen, and nothing matches. Then you might even get a coordinated care plan. One of our coordinators is doing a care planning for this one gentleman today, where there has already been a care planning meeting and he wasn’t supposed to get any home care. Now he’s gotten worse and will definitely need home care. […] So, it depends a bit on when in time it's done.*

*(Participant 25, registered nurse, municipal home care)*

However, if care planning is performed too late, the personnel at the receiving end will have limited time to arrange for the arrival at home. The timing of the functions also relates to the concept of synchronisation, i.e., agreement between the sending unit and the receiving unit. The analysis revealed the importance of having time and opportunity for preparation, so that everyone is ready when the patient is discharged. This includes both social care personnel, the patient and their family:

*Yeah, things move quickly once they’re … when you think that now such-and-such is done with treatment and should go home and then it’s just wham-bam…*

*(Participant 4, occupational therapist, municipal rehabilitation services)*

Additionally, the time of day when functions are performed can have major consequences for any workarounds that must be made to gain control over a situation and resolve it in the patient’s best interest. In particular, the weekend is a vulnerable time where the personnel must use ‘firefighting’ methods to meet care needs, until more permanent solutions can be put in place:

*We had one person come home on a Friday, who couldn’t get out of their wheelchair and did not have a wheelchair lift at home. And then we got a hold of … an emergency occupational therapist and physical therapist and had them bring a lift that they could borrow and use over the weekend. And then our occupational therapist and physical therapist had to come over on the Monday.*

*(Participant 37, assistant nurse, municipal social care)*

Friday night was a time that was frequently mentioned in the narratives of the participants as a known risk factor in discharges. During evenings and weekends, resources are reduced, with limited access to decision-makers and personnel with knowledge, existing relationships with patients and insight into the care planning processes, which were performed during weekdays. At best, the personnel might have access to written documentation, but this could also be limited during weekends if secretaries had not yet had the time to transcribe the physicians’ notes into the electronic patient records.

*If someone is discharged on a Friday afternoon and then there’s just temporary staff all weekend and then maybe, you’ll get a message about that on Monday or something and then maybe you’ll talk it over with the regular municipal personnel. But of course, things can happen over the weekend and probably do sometimes. And sometimes it’s not … sometimes it takes a long time before the patient discharge summary is transcribed and sometimes things there are missed. I think that’s a potential problem.*

*(Participant 22, physician in primary care)*

#### Precision

The precision of the performed functions relates to the quantity and quality of patient care. During a hospital stay, many units and professions are involved in the process of planning for sustainable continued care at home. The findings of the study suggest that gaps occur in this process, obscuring possible problems which can surface when the patient comes home. One such gap is the fact that the social care personnel with the closest relationships to the patient are neither invited nor informed first-hand regarding care needs during the discharge process. Another illustrative example relates to information transfer: too little information about a patient leads to knowledge gaps, creating extra work to gather the missing information. This may lead to unnecessary examinations or tests or deficient care. However, too much or irrelevant information can also create problems, as important information may be obscured.

*That the information is given, that it is relevant, a bit brief. If there’s too much text, you realise that you kind of lose track of what’s important. Straightforward, clear communication, with short pathways, that they aren’t too long.*

*(Participant 14, registered nurse, municipal home care)*

As emphasised in the quotation above, effective, clear and concise communication is of utmost importance for successful care transitions. The precision of information and communication regarding relevant issues is equally important for social care personnel and when informing the patient:

*The patient is just buried in information and paperwork when they are discharged from hospital, so they can’t remember what’s important. They get confused … it’s better if the important stuff is written down and then they can read it afterward.*

*(Participant 118, coordinator, municipal home care)*

The core issue of precision in care transitions may thus emerge in the quality of information transfer and the availability, procedure and use of systems where information is stored. Availability of information in multiple systems can be considered a strength, but can also generate extra work:

*Yeah, the systems are … I kind of think that you should have that information more available. There are so many different systems that you have to access to see it.*

*(Participant 13, registered nurse, municipal home care)*

There is an apparent gap in information transfer in the system, which can be illustrated by the case of ambulance personnel arriving at an older person’s home and trying to get the bigger picture of what the patient’s usual status was and what had been decided at hospital discharge. Access to written information was limited or non-existent, and there was difficulty getting in contact with municipal healthcare personnel with knowledge of the patient. The result was that the patient was returned to hospital and readmitted, but the solution could have been different:

*It’s so hard to get information about the patient … what has been decided … I’ll look through binders and call, but I feel like I never get any information, you just stand there with your papers and wonder … So that’s what’s missing, from the side of the municipality. I mean, if you’re supposed to call one of the municipal nurses, then the person in charge will have gone home or is off or it’s in the evening or there’s no binder and there aren’t any papers and I don’t have the medical records and I don’t have anything … a care plan or whatever it is that you’re supposed to … that’s been decided, and when I’m standing there and I don’t have any idea, then maybe we’ll load up that patient and head to the emergency room. And then they’ll do something … small or, yeah, they’ll admit the patient for a night or something and then it all starts over. So, you get unnecessary care and unnecessary trips for these kinds of patients.*

*(Participant 40, ambulance nurse)*

The precision of the care planning is also a central issue. Multiple stakeholders in various professions prepare a plan of action, together with the patient and family, regarding the care efforts needed when the patient gets home. However, such planning is hypothetical, since there are limited possibilities to know what everyday life will be like for the patient after discharge:

*The environment is different when you get home. The conditions are completely different.*

*(Participant 4, occupational therapist, municipal rehabilitation services)*

To counteract this, municipal care personnel would wait a few days to let the patient get acquainted with the care efforts and aid tools and have time to recover from the hospital visit:

*And then I’ll come back a day or two later and talk a bit more once they’ve settled in at home and when there’s hopefully a bit more peace and quiet […] get an idea of how things have actually gone during the first few days at home. That follow-up is valuable, I think, because then I can see quite a lot.*

*(Participant 1, registered nurse, municipal home care)*

### Management of possible occurrences of uncontrolled performance variability

In the final step of the FRAM analysis, we propose ways to manage possible occurrences of uncontrolled performance variability as presented above. The precision of functions related to the planning of resources was shown to vary greatly. This indicates that for healthcare providers to have the ability to act even in uncertain conditions, their possibilities for adaptation need to be increased. Functions like ‘Execute coordinated care at home’ have several prerequisites and controls originating from the output of the function ‘Execute care transition’. However, the analysis indicates that variability in precision can also be dealt with by increasing the flexibility in a patient’s home after discharge. Thus, one way to dampen the effects of functional resonance could be to redistribute resources and increase them in a patient’s home the first few days after homecoming and then perform regular and frequent reassessments, to adjust care flexibly and accurately. This would enable adaption to the care needs that emerge during the first few days as a way to manage uncontrolled performance variability.

The timing of the care transition also affects the possibility of responding to unexpected events, as the system appears to be more vulnerable during evenings and weekends. Variability in timing can mean that the patient is discharged too early or too late, causing a ripple effect and functional resonance with consequences for downstream functions. One way of managing this could be adding prerequisites and controls for the function ‘Execute care transition’ so that the time of discharge will not be during the vulnerable periods. The downstream function ‘Execute coordinated care at home’ requires some components supplied from the hospital – such as medical information regarding the care given, medications needed during the first few days, and an updated medication list – and some components from the municipal organisation, such as availability of adapted care aids for basic mobility and ergonomic work conditions for personnel. The most critical functions in the system for monitoring and directing efforts to manage variability are ‘Co-create plan of coordinated care’, ‘Prepare care transition to home’ and ‘Execute care transition’. These three functions together constitute the grounds for the care performed at home.

## Discussion

The study aimed to map coordination of care across healthcare and social care organisations, describe interdependencies and system variability in the discharge process for older people with complex care needs and evaluate the alignment of the discharge planning with the needs that arise at home. The modelling of the discharge process reveals, in line with former studies [27, 29, 30], that discharge planning is complex. There are multiple task interactions and numerous functional dependencies across organisations, with countless occasions where things can go wrong. As shown in a study of complex sociotechnical systems, a reductionist approach cannot provide a sufficiently detailed and holistic picture of the interacting components of a system to guide improvement [52]. The results of the current study demonstrated that the FRAM could help create an accurate and comprehensive view of the cross-organisational collaboration, making it possible to identify areas of improvement and changes that might lead to a more efficient and effective discharge process. The FRAM provided insight into the complexities of interdependencies and variability in the components involved, such as how individuals, teams, and organisational structures – as well as the technical aspects of the discharge process – interact and influence each other throughout the discharge process. This could be used to identify potential barriers and to set strategies to achieve better outcomes for patients.

This study adds to the knowledge from other studies using the FRAM, by increasing our understanding of the uncontrolled variability in the outcome of the discharge planning, suggesting that detailed planning for care at home is almost impossible. If viewed using the framework of WAx [17], WAI in this study would rely on work-as-prescribed and work-as-normative as presented in the guidelines and policy documents by blunt-end operators. While the policy documents presented hospital discharge as a linear process in eight consecutive steps, the FRAM model revealed a total of 52 dynamic functions to realise a transfer from hospital to home. In practice, the functions are often performed in parallel, are intertwined, or are performed in a back-and-forth, dynamic cooperation between a multitude of professions and stakeholders. These people work in separate documentation systems, with different technical solutions and access, regulated by different laws and regulations, which further increases complexity. Although the descriptions in WAI from ‘the blunt-end operators’ (i.e., managers and policymakers) [17] seek to give a direction and goal for the discharge process, following a standardised and normative protocol would be difficult – if at all desirable, given the diverse needs of and requirements for the patients being discharged from hospital to home. The findings reveal WAD to be inherently different from WAI, despite the best efforts of HSCPs. The differences in professional backgrounds, values, and cultures, along with the patients’ varying recovery processes, home environments, and supportive resources at home, mean that the reality is almost always different from the plan [53–55]. This study suggests that resources could be more efficiently utilised to manage possible occurrences of uncontrolled performance variability. Primarily, resources could be redistributed to allow flexible adjustments during the first few days after the return home, as some patients need less resources than planned while others need more.

Discrepancies between daily practice (WAD) and how the process is described in protocols (WAI) have also been found in other studies [41, 53, 54, 56–59]. A large discrepancy between WAI and WAD may be an indicator of brittleness in the system [60]. Thus, understanding why deviations occur is fundamental to improving patient safety [60, 61].

However, there is a fine line between adapting to varying conditions (i.e., being resilient) and going beyond the realm of safety due to the pressure of a high workload, limited resources, or the system's expectations of high efficiency [62]. The FRAM model highlights the limitations in the system design that lead to ‘workarounds’ in order to manage certain tasks in time and align WAD with WAI [59]. This study reveals several gaps arising during the discharge process, though these are not always visible until the patient faces reality at home, further emphasising the importance of flexibility and adaptability. The process mapped in WAD works because individuals go beyond their responsibilities and do more than expected to manage the negative effects of variability. The findings thus indicate that the discharge process as described in WAI is not adapted to the current healthcare system design, given existing staffing levels, structures, and information systems.

Furthermore, gaps occur in the information exchange between HSCPs in the planning and performance of care transitions. In the findings, the most prominent gap is that home care personnel, who often have the most up-to-date information about a patient, are not included in the information transfer to the same extent as other parties. Instead, they are supplied with certain information fragments after the patient has returned home [63]. Experiences of gaps in care are common, often arising from failures in communication and coordination of care [64] and interoperability of systems [65]. Cross-organisational bridges may mitigate such gaps in care [66]. The findings suggest that there are possibilities of creating shared documentation of a patient’s current care needs and plans. Previous research indicates that sharing of patient data across systems, if performed effectively, can create the basis for safe care [67, 68], while also reducing the workload for healthcare providers [69], limiting treatment delays [70] and decreasing overall costs for the healthcare system [71].

Collaboration involving multiple individuals and teams within and across organisational boundaries is expected during the transition process, even though not all individuals and teams communicate. If the healthcare team is seen as a CAS [72], healthcare professionals need to expand the system boundaries to understand how any action taken (or not taken) in their part of the system has a ripple effect throughout the entire system. Healthcare professionals in hospital care must anticipate what problems HSCPs at the receiving end will have when the patient is discharged. In their turn, professionals in home healthcare need to manage adaptations and moderation of goals when care plans ‘as imagined’ do not fit with the needs in the patient’s home. Systems thinking and anticipation of the consequences of processes across departments remain major challenges [73].

As regards the issues of timing in the discharge process, the study shows that the time of day when the patient is discharged strongly affects the ability of personnel to deal with unexpected situations. Friday afternoons are mentioned as a particularly vulnerable time for discharge of people with complex care needs. Previous studies have shown that the time of day when a patient is considered medically fit sets temporal conditions and time pressures for subsequent actions [29]. Decisions later in the day were associated with increased time pressure and variability, thus affecting precision. Other studies indicate that the time between the decision on discharge and the care transition is a potential barrier to information sharing, as time constraints lead to greater time pressure, increased performance demands and decreased flexibility [74]. Complex systems are resilient when they have diversity, as different professionals deploy different behaviours to respond to actions and situations based on evidence and their own experiences [75]. However, according to complexity theory, a system can never be fully described or fully controlled [76]. This creates challenges in care transitions since the findings suggest many interdependencies between the actors involved, meaning that they depend on each other and on collaboration. Rasmussen [77] emphasises how circumstances may not be under the control of any one person, and that there may be systematic but unseen traps set by upstream decisions.

### Strengths and limitations

Care transitions for older people with complex care needs involve multiple stakeholders and a diversity of professionals and organisations. Furthermore, the system’s adaptive complexity makes it difficult to overview, study, and evaluate. The FRAM showed potential to uncover the complexity in collaborative work in the dynamic discharge process, which can unfold in a variety of ways. This might have been difficult to do with other methods. The FRAM allowed for a visualisation of the everyday activities in the interprofessional team collaboration across organisational borders and the roles of the different professionals during the discharge process. In this study, the FRAM offered a thick description of a process that normally remains hidden and thus enabled potential development of new guidelines. Further, it also provided a detailed model of WAD and performance variability of the complex coordination of transitional care across care providers, which can be used as a guide for improvements. However, using the FRAM requires expert knowledge and experience along with access to the research field. Therefore, the method cannot be used for all types of processes [39] and may be too resource-intensive and demanding for use in healthcare quality improvement generally.

A key strength of this study was inclusion of multiple perspectives across a variety of healthcare settings to explore how different clinical teams successfully support care transitions. However, the heterogeneous sample may have limited the ability to achieve theoretical saturation. On the other hand, we were not looking for a representative sample, but one that encompassed participants with as broad knowledge, skills, and expertise as needed to answer the research question [78, 79]. The number of participants is related to concerns about the richness and thickness of the data [79]. Thus, the large amount of material represents a strength in this study. Multiple data collection methods were used as triangulation enables qualitative researchers to study relatively complex entities or phenomena in a holistic way [80]. The study was performed over the course of more than a year. To reduce a possible ‘Hawthorne effect’ in observations [35], the researchers emphasised that it was not the performance of individuals that was being observed and that the participants were not in any way being assessed in their work. Furthermore, the observer was dressed in hospital scrubs, like other healthcare personnel, to blend in.

Constructing the FRAM model was a trade-off between showing all the related functions and achieving a usable and understandable model. Although in-depth information was obtained on each function, some perspectives to create a complete picture of the discharge process may be lacking. To mitigate this, the model was checked for sense by professionals with experience of working with older people with complex care needs and the care transitions involved. This expert audit was performed to increase ecological validity.

This study was performed in a single region of southern Sweden and the sampling in hospital was limited to medical and geriatric wards, to gather data that were general across the different geographical locations. Future studies should be performed in other settings to elucidate whether the results may be transferred. The study aimed at mapping and describing intra- and inter-organisational processes with a focus on coordination and collaboration between different professionals and stakeholders. This study contributed to increased understanding of the system of hospital discharge and care transitions from the perspective of healthcare professionals. It did not include patients, family, or other informal caregivers. We want to emphasise the importance of including patients and families in future studies [15, 81].

### Implications for practice

This study provides a comprehensive visualisation of the most prominent gaps and vulnerabilities in the care transition process indicating where special attention to communication and coordination is needed. The findings show the importance of consensus in the care planning process and that all relevant information is collected, distributed and made available to the parties involved in the decision process and particularly in patient care at home. Reinforcing the ability to bridge gaps through collaboration may improve safety [82]. However, the possibility of bridging the gaps depends on both access to information and inclusion in the process. During the hospital stay, possible problems or issues waiting at home do not always surface, making the planning of care at home a hypothetical reality, left for HSCPs in the patient’s home to deal with after discharge. To achieve a safe and secure discharge and subsequent care at home, proactive work during the discharge planning process needs to be facilitated. This may be done by increasing precision in the assessment and planning of a patient’s care. Since this is a time-sensitive process, considerations need to be made as to at what time and on what day of the week the discharge will be performed, so the variable needs can be dealt with accordingly. It could be useful to have a clear definition of what a secure discharge for patients with complex care needs should be, and what parameters need to be fulfilled. If security is intentionally created, the discharge process can proceed and perform successfully. Furthermore, to ensure the ability to respond to unexpected events and variations by adapting care efforts at home, a redistribution of resources during the first few days after the return home would increase flexibility and patient safety.

By promoting insight and knowledge of other HSCPs’ areas and creating the basis for a resilient organisation with HSCPs that anticipate and adapt by going outside their boundaries, we could increase the possibility of safely bridging the gaps in the system.

## Conclusions

The transition process from hospital to home for older people is complex, encompassing couplings and interdependencies in the exchange of information between various individuals, teams, and organisations. The FRAM-based approach revealed barriers and cracks in team collaborations. Discharge planning was carried out within a system that lacked control because of limited access to patient information and complexity in the communication channels between different organizations. Due to the varying health conditions of patients, and as their complex needs and requirements are difficult to predict and plan for, a mismatch between discharge planning and the needs at home may occur. This makes the arrival at home the most vulnerable point of the transition process. To optimise the outcome of the discharge process for patients with complex needs, the system’s variability and adaptations should not be stifled. Instead, the study suggests, the system might be kept stable and resilient through flexibility, adjustments, and allocation of adequate resources at the time of the patient’s return home.

The findings of this study can inform managers and other decision-makers on how coordinated care is designed and managed in care transitions to align with the varying needs in patients’ homes after discharge. It can serve as a roadmap to future studies regarding the development of guidelines and training, as well as multidisciplinary simulations to improve intra- and inter-organisational collaboration and ultimately improve care for people with complex care needs.

### Supplementary Information

**Additional file 1. **

## Data Availability

The datasets generated and analysed in the current study are not publicly available, to protect the privacy of the participants. Qualitative interview transcripts could be used to identify participants and cannot be shared publicly as per the Research Ethics Board’s guidelines.

## Abbreviations

CAS: Complex adaptive system
FRAM: Functional Resonance Analysis Method
HSCP: Healthcare and social care professional
WAD: Work-as-done
WAI: Work-as-imagined
