# Supplementary material for: Exploring interdependencies, vulnerabilities, gaps and bridges in care transitions of patients with complex care needs using the Functional Resonance Analysis Method
Source: BMC Health Serv Res. 2023 Aug 11;23:851. doi: 10.1186/s12913-023-09832-7 (PMC10422836; doi:10.1186/s12913-023-09832-7)
Supplement: Supplementary file 1 — Additional file 1. [file 12913_2023_9832_MOESM1_ESM.docx]

# Interview guide (translated from Swedish)

The overall aim of the thesis work is to investigate care transitions by exploring how integrated care and a cohesive care chain can maintain good and safe care for older people in need of coordinated care.

We start from the assumption that professionals involved in care or social care of older people in need of coordinated care have valuable experience and knowledge. We want to gather and compile this knowledge, to highlight good examples but also to find possible obstacles or difficulties that may need attention.

The term "elderly in need of coordinated care" is used in this study to refer to an elderly person (aged over 65 years, but more often over 75 years) who has several concomitant diseases or disabilities and has interventions from both home care and social care. Similar concepts are frail elderly or elderly people with complex care needs. It is this group of patients that we refer to in the rest of the interview, even if we say only "elderly".

There are no right or wrong answers to the questions. We want you to freely describe how you think and all answers are welcome.

Participation is voluntary. You can choose to end the interview at any time. All results will be presented at the group level and it will not be possible to link any quotes to specific individuals.

To facilitate the analysis work, the interview will be recorded. Is that okay with you?

I will now start the recording.

[START RECORDING]

Today's date is XXXX and an interview is taking place with informant X.

And I will repeat the question, is it okay for me to record this?

## Introduction

Describe your role and function in the care chain of the elderly person. What does your work entail?

Can you describe what safe and secure care throughout the care chain for the elderly means? How can it be created through everyday healthcare? How do you work to make this happen?

What do you see as important for you/your colleagues/healthcare as a whole to be able to provide good treatment and care to the elderly?

## Discharge from hospital

1. Tell us what happens when an elderly person has been treated in hospital and is ready for discharge to the home, with a need for interventions in the form of both medical and social care. Where do you come in?
2. What information do you receive? How? (from whom, through calls, systems etc.)
3. What do you need to know? What information is missing?
4. The planning that has been done inside the hospital, how do you feel it works when the patient/care recipient has returned home? Is the plan sustainable? Why, why not?
5. What benefits do you see resulting from the patient's care plan? What knowledge about the plan is needed? Do you have access to the plan today?
6. Can you describe an example of a poorly functioning collaboration and what results this had? Can you describe an example of a working collaboration?

What would be different in what you have told us if the person was already known to you and was coming home with altered needs?

## Coordination/collaboration

How do you coordinate or collaborate with other professions or care providers around older people? What professions or care providers do you interact with?

When is collaboration/x relevant? How does it work?

What gaps or risks do you see? How can they be bridged?

Who has a holistic view of an elderly person's situation?

## Conclusion

What are the main difficulties/obstacles in creating safe and secure care throughout the care chain for older people? Based on your experience, what are the weak links in the care chain for the elderly?

What are the most important things that contribute to creating safe and good care throughout the care chain for the elderly? How could the current system be changed to achieve that? What changes do you see as necessary?

## Lastly...

Is there anything you'd like to add?

[END RECORDING]
